# Inequity in care delivery in cardio-oncology: dissecting disparities in underrepresented populations

**DOI:** 10.3389/fonc.2023.1124447

**Published:** 2023-06-09

**Authors:** Shruti Rajesh Patel, Giselle Alexandra Suero-Abreu, Angela Ai, Maya K. Ramachandran, Kelly Meza, Narjust Florez

**Affiliations:** ^1^ Department of Medicine, Division of Oncology, Stanford University and Stanford Cancer Institute, Stanford, CA, United States; ^2^ Massachusetts General Hospital, Harvard Medical School, Boston, MA, United States; ^3^ Olive View-University of California, Los Angeles Medical Center, Los Angeles, CA, United States; ^4^ Dana Farber Cancer Institute, Boston, MA, United States

**Keywords:** equity, cancer, cardiology, cardiooncology, cardiotoxicity, oncology

## Abstract

It is well known that patients with cancer have a significantly higher cardiovascular mortality risk than the general population. Cardio-oncology has emerged to focus on these issues including risk reduction, detection, monitoring, and treatment of cardiovascular disease or complications in patients with cancer. The rapid advances in early detection and drug development in oncology, along with socioeconomic differences, racial inequities, lack of support, and barriers to accessing quality medical care, have created disparities in various marginalized populations. In this review, we will discuss the factors contributing to disparities in cardio-oncologic care in distinct populations, including Hispanic/Latinx, Black, Asian and Pacific Islander, indigenous populations, sex and gender minorities, and immigrants. Some factors that contribute to differences in outcomes in cardio-oncology include the prevalence of cancer screening rates, genetic cardiac/oncologic risk factors, cultural stressors, tobacco exposure rates, and physical inactivity. We will also discuss the barriers to cardio-oncologic care in these communities from the racial and socioeconomic context. Appropriate and timely cardiovascular and cancer care in minority groups is a critical component in addressing these disparities, and there need to be urgent efforts to address this widening gap.

## 1 Introduction

A diagnosis of cancer, irrespective of the primary cancer site, is associated with an increased risk for cardiovascular death and nonfatal morbidity (1–3). Among cancer patients and survivors, cardiovascular disease (CVD) is the primary cause of death, and patients with cancer have 2-6 times higher cardiovascular (CV) mortality risk than the general population (4). A recent study among more than 7.5 million cancer patients showed that CVD contributed to 5.24% of deaths among all cancer patients with a heart disease-specific mortality rate of 10.61/10,000-person-years. In addition, the mortality ratio of fatal heart disease among all cancer patient studied was 2.24 times that of the general population, with variability due to age, race, primary cancer type, and follow-up time (2).

Similarly, prior large retrospective studies based on the Surveillance, Epidemiology, and End Results (SEER) database have characterized CVD mortality risk in cancer patients. A study with more than 3 million patients, including 28 cancers over 40 years, found that 11.3% died from CVD. The risk of CV mortality was highest in patients diagnosed <35 years and within the first year after a cancer diagnosis. Furthermore, the mortality risk remained elevated through follow-up compared to the general population (5). Similarly, a subsequent SEER-based study with more than one million patients diagnosed with breast cancer over 17 years found a 4.6% incidence of fatal heart disease, which increased at longer follow-up comprising up to 28% of deaths from non-primary cancer at 10 years (6). Another interesting study used SEER data in nearly 5 million patients over 14 years and showed that the higher rate of cardiac death for cancer patients is not uniform in all patients and is higher for non-white ethnic groups. Specifically, the risk of cardiac death in cancer patients was 1.16% higher than in the general population, but when stratified by ethnicity, the risk was 1.76, 2.28, 3.68, 2.65, and 1.84 for Whites, Blacks, American Indians/Alaska Natives, Asians/Pacific Islanders, and Hispanic/Latinx, respectively (7). These observations highlight the elevated risk of CV mortality from the point of a cancer diagnosis into survivorship and the need for earlier and more aggressive CV care in cancer patients. As such, the field of cardio-oncology has rapidly expanded in the United States (US) and globally to address the increased heart-specific mortality risk in cancer patients.

Cardiac complications of cancer therapy include myocardial dysfunction, coronary artery disease or peripheral vascular disease, valvular disease, arrhythmias, arterial hypertension, and thromboembolism (8). These cardiotoxicities have been associated with many categories of cancer therapy and relevant data on disparities in treatment-associated cardiotoxicities are described when affecting specific populations in subsequent sections of this review. Cancer and CV disease are linked, not only through the deleterious effects of oncologic treatments on CV health but also due to common risk factors, including age, obesity, diet, alcohol, and physical activity (9, 10). Over the past decade, the range of CV toxicities has expanded due to the introduction and rapid uptake of numerous targeted therapies, immune checkpoint inhibitors, and antibody-drug conjugates (11). As the overlap between heart disease and cancer patients continues to increase, the emerging field of cardio-oncology aims to identify patients at risk of CV complications related to cancer treatments, provide early detection and intervention for CVD, and develop strategies to prevent or minimize these complications. By addressing CV risk factors and managing CVD, cancer patients may have better outcomes and quality of life. Several professional organizations, including the American College of Cardiology, the American Society of Clinical Oncology, and the European Society of Cardiology, have recognized the importance of cardio-oncology and have developed guidelines and recommendations for managing CVD in cancer patients (12, 13). Despite the field’s rapid growth, there is a need to improve access to care at the local, state, and national levels and to underrepresented populations. Furthermore, there is a lack of resources about cardio-oncology within community-based oncology practices, thus, expanding into these areas is critical to provide care to significant segments of the cancer population (14).

The most common CV diagnoses in patients with cancer are hypertension (HTN), coronary artery disease (CAD), heart failure (HF), and arrhythmias. The incidence of these specific diseases in cancer patients varies depending on several factors, including the type of cancer, cancer treatment received, and preexisting CV risk factors. HTN is a common occurrence in cancer patients, with estimates of around 38% in cancer populations compared with approximately 26% of the general population (15–17). The incidence of developing CAD in cancer patients is increased compared to the general population and highest in the first six months after the initial cancer diagnosis (18–20). A study compared patients treated for breast cancer or lymphoma to age-matched controls and found that within 5 years of their cancer diagnosis, the risk of HF was 3x higher than in people without cancer. Furthermore, 10% of the survivors developed HF within 20 years compared with 6% of control subjects (21). A large-scale study assessing the bleeding risk of anticoagulation in patients with cancer found an incidence of ~20% compared to the prevalence known in the US between 1-2% (22, 23).

It is known that certain patient groups face unique challenges related to these cardiotoxicities and access to specialized cardio-oncology services. Special clinical and research efforts have been made in studying long-term CV risks of survivors of childhood cancers and monitoring the effect of this heightened risk on their CV health outcomes in adulthood. Care of elderly patients with cancer is also a unique challenge given their higher rate of co-morbidities which increase their risk of developing cancer treatment-related cardiotoxicities (24). Notably, little is known about how racial and ethnic disparities alongside structural, economic, and socioenvironmental factors impact cardio-oncology care. Particularly, access to appropriate and timely CV and cancer care by minority groups is a key element driving persistent disparities in cardio-oncology care. Existing evidence suggests that socioeconomic inequality affects the incidence, treatments, and outcomes of patients with cancer and CVD. Furthermore, a recent study highlighted the impact of social vulnerability on mortality rates in cardio-oncology patients showing worse outcomes in counties with greater social vulnerability (25, 26).

**Figure 1:**
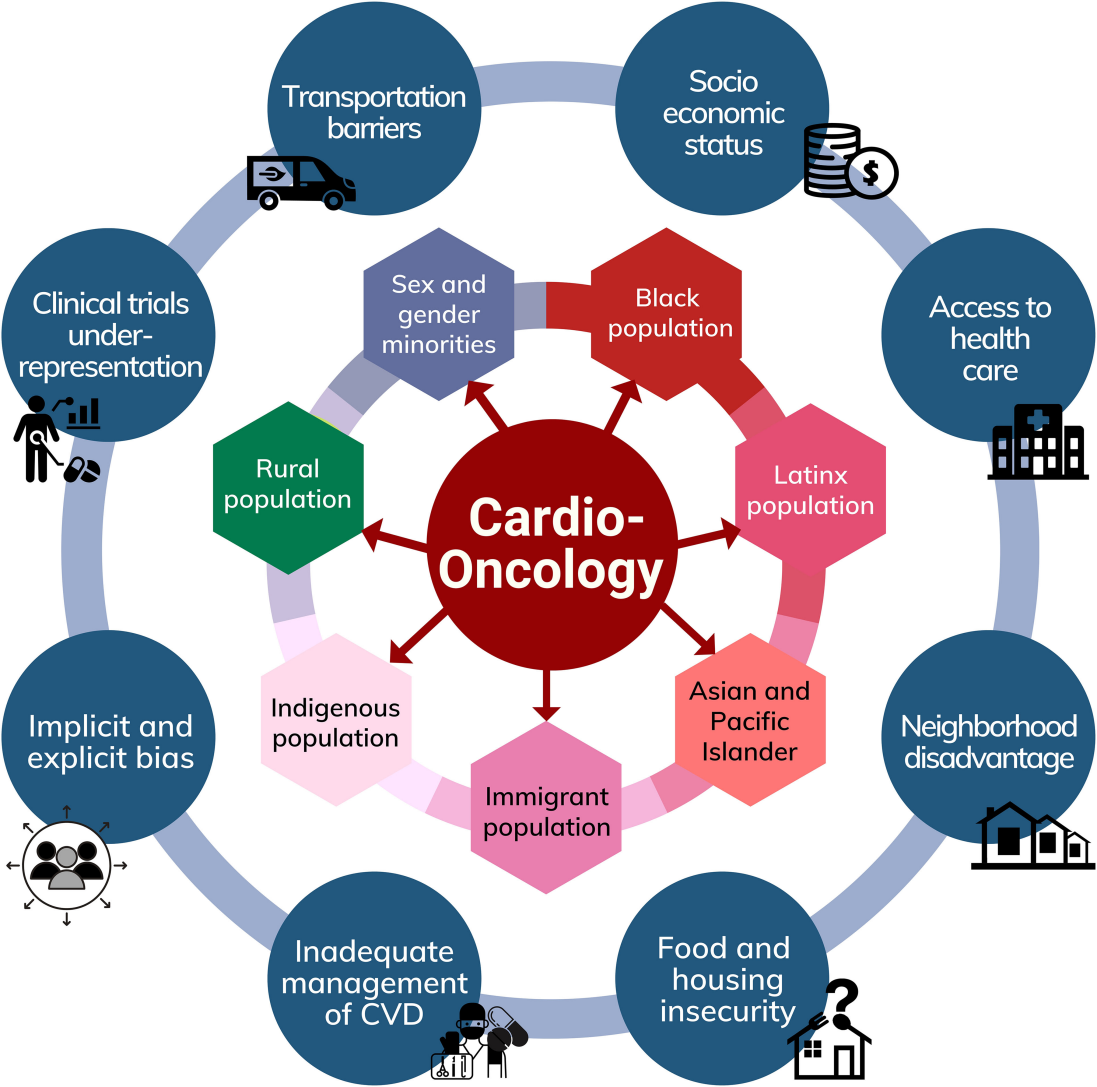
Factors contributing to disparities in Cardio-Oncology and populations at risk.

In this review, we will discuss cardio-oncological disparities in a variety of marginalized populations, including Black, Hispanic/Latinx, Asian and Pacific Islander (AAPI), indigenous populations, sex and gender minorities (SGM), rural populations, and immigrants. These populations at risk and distinct factors contributing to disparities in cardio-oncology care are outlined in **Figure 1**. We will expand on disparities that affect these minority groups, including the prevalence of cancer screening rates, cardiometabolic and genetic risk factors, and cultural factors. We will also discuss the barriers to cardio-oncologic care in these communities arising from the racial and socioeconomic context.

There is a known association between pre-treatment CV risk factors and post-treatment cardiac dysfunction, and it is imperative to discuss these disparities to understand how it affects cardio-oncology care (27). While the prevalence of CV disease has been declining in non-Hispanic white (NHW) populations, these rates remain stable in Hispanic/Latinx, Asian, and Black population (28). The COVID-19 pandemic in the US has led to a significant increase in deaths caused by heart disease and cerebrovascular disease especially among Black, Hispanic/Latinx, and Asian population. This suggests that these groups have been disproportionately affected by the pandemic’s indirect effects (29). In Hispanic/Latinx populations, cancer is one of the leading causes of death, and these patients are diagnosed with more advanced stages of breast, lung, and colorectal cancers compared with NHW individuals (30).

Irrespective of a biological difference, these disparities in cancer outcomes are also largely influenced by structural factors such as lack of insurance, transportation issues, decreased educational attainment, financial security, and less access to high-quality preventative care or specialized services (31–33). Furthermore, these patients do not have equal access to novel, high-quality therapies and are consistently underrepresented in clinical trials (34–36). In addition, the COVID-19 pandemic delayed the diagnosis and treatment of cardiac conditions and cancer, with implications that are still unclear to date. Importantly, it also highlighted the association of race and ethnicity–based disparities in CV and cancer care delays, medical care disruptions, and concerns about crucial socioeconomic factors alongside systemic and structural racism (37).

Despite significant advances in early diagnosis, risk factor mitigation, and drug development in the cutting-edge fields of cardiology and oncology, inequities in the structural, economic, and environmental systems continue to contribute to the long-standing higher prevalence and worse outcomes due to CVD and cancer care that consequently underlie disparities in cardio-oncology care (38). We present an overview of these disparities in cardio-oncology care from the viewpoint of special populations to raise awareness of the urgent efforts needed to improve the outcomes of these patients.

## 2 Cardio-oncology disparities in the Hispanic/Latinx population

The Hispanic/Latinx population constitutes nearly 20% of the US population and is the second largest racial/ethnic group. Despite a considerable underestimation in the 2020 census data, the Hispanic/Latinx population reached 62 million (39). Cancer and CVD are the two leading causes of death for Hispanic/Latinx individuals in the US (40). According to the American Cancer Society, there were 43,079 deaths due to cancer and 41,794 deaths due to heart disease among the Hispanic/Latinx population in 2019, representing 20% of total deaths (30). The mortality rates due to cancer and CVD among the Hispanic/Latinx population are impacted by social determinants of health (SDOH) and by challenges in immigration status, lack of health insurance, and healthcare bias (25, 41–44). Approximately 18 million foreign-born Hispanic/Latinx adults reside in the US, with about two-thirds being noncitizens and/or undocumented (45). Non-citizens and undocumented immigrants may not be eligible for health insurance and may lack employment opportunities that could offer it. Consequently, Hispanic/Latinx people represent a large amount of the US uninsured population at 30.1%, compared with the NHW population at 11.1% (39). Therefore, disparities in cardio-oncology care in Hispanic/Latinx patients partly stem from the impact of increased social vulnerability, low socioeconomic status, and lack of insurance, which causes barriers to accessing care and receiving timely preventive cancer and CV interventions.

For instance, there are known reduced screening rates in the Hispanic/Latinx population and, consequently, delayed diagnosis of preventable cancer such as lung, breast, and colorectal compared to NHW people (30, 44). This sometimes translates into advanced cancer stages at diagnosis and ineligibility for novel, less cardiotoxic regimens with a higher risk of cardiac dysfunction and worse patient outcomes (30, 38, 46, 47). Based on the American Heart Association report on heart disease and stroke statistics for the Hispanic/Latinx population in 2021, 52.3% of males and 42.7% of females had CVD alongside an increased burden of risk factors such as obesity (>78%), hyperlipidemia, (37%), hypertension (>40%), physician-diagnosed diabetes (15%), lifetime tobacco use (52%) and sedentarism (35%) (48, 49). For example, Puerto Ricans and Mexican individuals have more than twice the prevalence of diabetes mellitus compared with NHWs (50). Despite having higher rates of CV risk factors, the Hispanic/Latinx population in the US has lower rates of CV mortality compared to NHW Americans. The reasons for this paradox are not entirely clear, but some potential explanations include protective cultural and social factors, such as strong family ties and support, and healthy dietary habits (27). However, recent studies focused on the effect of neighborhood segregation and CVD among Hispanic/Latinx showed that county-level Hispanic/Latinx ethnic density is associated with increased CVD mortality. It also linked these areas to higher rates of uninsured individuals, fewer primary care physicians, and other adverse environmental factors (42). Similarly, in a large US population-based study, Hispanic/Latinx adults <45 years of age had higher mortality due to comorbid cancer and CVD than for either disease alone in counties with greater social vulnerability (25).

Unfortunately, there is a lack of data specific to the Hispanic/Latinx population regarding cardiotoxicity from cancer-directed therapies, and this is particularly disturbing as cancer and CV disease often co-exist in the same individual alongside other complex comorbidities (51, 52). An important element that limits our knowledge of cardiotoxicity risk specific to Hispanic/Latinx patients is that most studies report the Hispanic/Latinx population using aggregated data, which masks risk profiles and affects the accuracy of the results given their diverse demographics. More than 33 million Hispanics/Latinx in the US report two or more races in origin (53). However, this heterogeneity is not reflected in clinical studies, tailored therapies, or research on specific disease outcomes. Hispanic/Latinx patients are typically classified as a single group without distinction to their heritage, socioeconomic status, immigration pattern, and cultural characteristics. Thus, standardizing clinical care and research with disaggregation of Hispanic/Latinx subgroups will be critical to further understanding the cardiotoxicity risk profile within these groups. Another area to be further studied is the implication of specific cultural characteristics shared across the Hispanic/Latinx subgroups, such as *familismo*, *personalismo*, and strong religious values that tend to influence health behaviors and outcomes (46). For example, studies suggest that residence in close communities and *familismo*, where family members are a vital source of support with health issues, may explain why the Hispanic/Latinx population has lower mortality despite the described poor baseline cardiometabolic risk profiles (54–56). However, data shows that these cultural factors may be offset by both acculturation and duration of residence in the US, which have been associated with a negative impact on CV outcomes as more people assimilate US behaviors and diets, which may further increase the risk profile for these patients (42, 56).

There is also a chronic underrepresentation of Hispanic/Latinx people in clinical trials in cardiology and oncology (57, 58). This limits our understanding of potential biological differences related to cardiotoxicity in these populations and delays efforts for individualized care in the era of precision medicine. The lack of representation within clinical trial also delays patients ability to receive newer therapies increasing the differences in overall survival between the populations. Limited enrollment in clinical trials is attributed to several factors such as linguistic barriers, limited understanding of treatment options, inability to navigate the complex medical system, difficulties with the informed consent process, distrust of the health system, physician bias, structural racism, poor communication with their physicians, and financial concerns related to the logistical burden of trial participation (59, 60). There is a need for an intentional effort to improve Hispanic/Latinx representation in cardio-oncology trials and to promote diversification of the clinical trial workforce and leadership to increase diversity and equity in the field.

## 3 Cardio-oncology disparities in the Black population

The Black population is the third largest racial group in the US and represents 13.6% of the population. Black patients face significant obstacles in cancer risk reduction, early detection, and treatment and typically are diagnosed with a higher tumor burden and advanced stage. Additionally, for most cancers, Black patients have the shortest survival and highest rate of death compared to any other racial/ethnic group (61). Currently, the largest disparity in Black patients exists in uterine, stomach, prostate, and plasma cell cancers, for which death rates are twice as high in Black people (61). Black individuals are known to have earlier onset of traditional CV risk factors due to a variety of systemic and biological factors that increases the incidence of HF, stroke and peripheral vascular disease (62). The higher prevalence of CVD linked to poorer access to primary care (63), which is ultimately linked to the fact that Black populations face a disproportionate amount of adverse social and environmental characteristics in the US. This disparity in pre-treatment cardiac function further increases the risk of cardiac dysfunction with anti-cancer therapies and patient been started in cardiotoxic therapies without prior CVD evaluation or risk assessment (64). Based on the national US cancer database, Black women with breast cancer were at a 25% greater risk of CV death compared to NHW women (65). Differences in underlying CV disease risk contribute to the difference in mortality risk, in addition to other known contributors such as social determinants of health, as Black women with breast cancer are 40% more likely to die than NHW women (66, 67). Black women also have a higher risk of triple negative breast cancer which leads to higher rates of chemotherapy and radiation, which further increases CV risk. Furthermore, Black patients with breast cancer experience more significant psychosocial stress from unmet informational, financial, and practical needs. The perceived discrimination and racism experienced by Black women have also been shown to contribute to low-grade chronic inflammation and CV disease (68).

A majority of the data on cardiotoxicity historically has been with anthracyclines and trastuzumab, although there is a growing interest in the cardio-oncology community to understand the cardiotoxicity of the newer targeted agents. A historical retrospective dataset demonstrated that Black patients treated with doxorubicin had a 3-fold higher risk of cardiotoxicity compared with non-Black patients (69). Furthermore, another study of patients with breast cancer demonstrated that Black women were greater than 2 times more likely to develop trastuzumab-related cardiotoxicity compared to NHW women, even after controlling for baseline CV risk factors (70). Furthermore, a meta-analysis across North America and European patient populations demonstrated that Black race was an independent predictor, similar to CV risk factors, of clinical and subclinical cardiotoxicity in breast cancer (71). Most of the prior cardio-oncology research in Black patients has focused on breast cancer in which clear disparities have been demonstrated, further research in other tumor types is necessary, as well as long-term follow-up data in this high-risk population. In addition to expanding research, strategies to assess and intervene in patient’s needs with the addition of interdisciplinary resources are critical

## 4 Cardio-oncology disparities in the Asian American and Pacific Islander population

The Asian American and Pacific Islander population (AAPI) is one of the fastest-growing racial groups in the US, with a 35.5% increase in population since the 2010 census (72). While often grouped, the term Asian American encompasses a wide range of ethnicities and racial groups and is remarkably heterogeneous. In recent years, there has been a push to disaggregate data related to the Asian Pacific Islander community, as it results in misleading inflation of survival statistics for this population (65, 73–76). Currently, major registries aggregate cancer data from Asian, Native Hawaiian, and Other Pacific Islander (NHOPI) and other Asian American populations, so we will cite data as initially collected in this section. Compared to individuals from another racial or ethnic group, Asian Americans have the lowest rate of developing cancer; however, cancer is the leading cause of death for Asians in the US (73). It was found that even when census tract poverty rates are accounted for AAPI men have a lower 5-year survival rate than NHW (58). Asian Americans are disproportionately affected by cancers because of infectious origins (ex. Hepatitis-B related liver cancer) and have the highest lung cancer rates among never-smoking women (77). Regarding CV comorbidities among the AAPI community, few studies have examined the subgroups separately (78). Generally, when looking at traditional CVD risk factors, it was found that there are associations similar to those reported in NHW Americans (79), however, there is known discordance between CV risk estimates depending on racial/ethnic groups (78).

Regarding cardio-oncology specifically, Asian American and NHOPI individuals are a small percentage of clinical trial participants, which limits the information regarding side effects and toxicities in this population. In a meta-analysis of randomized control trials for CV disease, of the 45 trials identified, only 11 reported race; of that 11, only 4 of those trials reported Asian American inclusion, ranging from 1.4% to 5% (80). A recent study showed that AAPI, and Hispanic/Latinx people, had the highest relative increase in cardio-oncology mortality between the 4th and 1st social vulnerability index (SVI) quartiles compared to the other population studies (25). It is clear that further research regarding the intersection must be done.

## 5 Cardio-oncology disparities in the Indigenous population

The Indigenous population in the US comprises approximately 9.7 million people and is incredibly diverse, with 574 federally recognized tribes and more than 200 unrecognized tribes (39). This population has the highest racial misclassification in health data compared to other groups in the US, so any disparities are likely an underestimation (81). It is essential to put the health of the Indigenous population in the US, as with all minority groups, into a historical context. European colonization and policies on the national and state level have all contributed to the existing health disparities in the Indigenous population, which have led to large health disparities (82, 83). Cancer disproportionately affects the Indigenous population in the US (65, 84, 85). Interestingly, there are differences in cancer risk and disparities seen when comparing Indigenous people living in different regions of the US. For example, compared to the White population, the incidence rate for all cancers combined is 23% lower in the American Indian/Alaska Native (AI/AN) population living in the Southwest but 49 percent higher in those living in the Southern Plains (86).

CVD is the second leading cause of death for the Indigenous population in the US (supplanted by COVID-19 in recent years), with over a third of CVD-related deaths occurring before the age of 65 (87). One study found that among a study population of almost 100,000 Native Americans, the prevalence of peripheral arterial disease in indigenous Americans was nearly twice the rate compared to NHW, even when controlling for atherosclerotic risk factors (88). This inequity is further exacerbated by the fact that hospitals in areas that serve indigenous populations often lack specialized services such as cardio-oncology. A recent scientific statement from the American Heart Association emphasized the need and importance of future studies and interventions to reduce and eliminate inequities faced by the Indigenous population of the US (89).

## 6 Cardio-oncology disparities in sex and gender minorities

The number of individuals identifying as part of a sexual and gender identity minority (SGM) is growing. The most recent projections estimate roughly 7.1% of the US adult and pediatric population identifying as lesbian, gay, bisexual, or trans* (LGBT), up from 3.5% in 2012 (90). Given the stigma associated with identifying as SGM and structural inequalities, it is often thought that these numbers are an underestimation of the actual population of SGM in the US (91). Unfortunately, data regarding cancer incidence, outcomes, and treatment responses for SGM people is sparse. Similarly, there is a large gap in understanding CV disease relating to the SGM community (92). Despite the paucity of research, it is clear that SGM populations face disparities relating to care due to experiencing multiple barriers to receiving health care (93). For example, lesbian and bisexual females are more likely to have difficulty accessing care with a regular provider than heterosexual females (94). Other potential barriers to adequate health care for LGB individuals are implicit bias and overt discrimination during health care encounters, which may lower trust in health care providers and the health care system (94, 95).

There are known cancer disparities in SGM communities, which include increased rates of melanoma in cisgender gay men and increased rates of Kaposi sarcoma, lymphomas, and anal cancer in those populations at increased risk for HIV infection, including cisgender gay men and transgender women (96). Additionally, as cross-sex hormones administered for gender affirmation may be delivered at high doses over decades, the carcinogenicity and cardiotoxicity of hormonal therapy in transgender people is an area of continued research. Outside of these specific disparities, however, there is still much to learn.

Similarly, there is evidence that within the CV disease space, there are disparities related to the LGBTQ population, which was recently called to attention by the American Heart Association. When examining risk factors for CV health, sexual minority women exhibited greater CVD risk related to tobacco use, alcohol consumption, illicit drug use, poor mental health, and body mass index. In contrast, sexual minority men experienced excess risk related to tobacco use, illicit drug use, and poor mental health (97, 98). While the SGM group has often been examined as a monolith, there have been increased efforts to parse out variations in CVD risk by the sex assigned at birth, gender identity, sexual orientation, and race (92). One area of interest is the relationship between gender-affirming therapy and CVD (99). When examining from a lens of cardio-oncology space, minimal data exists, and further research is needed to expand appropriate care to this expanding population.

## 7 The impact of immigration status on disparities in immigrant populations

The term immigrant is defined as a person who comes to a country to establish permanent residence. In the U.S. immigration law, “immigrant” means explicitly those inspected and admitted as lawful permanent residents (100). Notably, this technical definition may underestimate population-data analysis of the total foreign-born population present in the US based on limited information on the legal status. Typically, it does not include the institutionalized population, which is primarily people in nursing homes and prisons. Immigrants will be denoted in this paper based on the Census Bureau definition, including three principal legal-status groups (naturalized citizens, legal permanent residents, and undocumented immigrants). Based on the Census Bureau’s monthly Current Population Survey (CPS) by the Center for Immigration Studies, the total foreign-born or immigrant population in the US reached 47.9 million in September 2022. This represents 14.6 percent of the US population or one in seven US residents and an increase of 2.9 million since January 2021. This is also one of the largest numbers in the US government census compared to the high records reached in 1890 and 1910 (101). Immigrants are considered a vulnerable population, but there is heterogeneity among the different ethnic and socioeconomic groups and language barriers, which relates to the degree to which they are vulnerable to inadequate health care (100). Most of the studies on immigrants and health care have focused on Hispanic/Latinx people as one of the largest immigrant groups, followed by Asians (a term that masks great ethnic diversity) and, more recently, on Black and Black/Caribbean immigrants. Across these groups, many similar factors influence inadequate health care in immigrant populations affecting their CV and cancer care and consequently increases disparities in cardio-oncology (102, 103). These factors include socioeconomic background; immigration status; food and housing insecurity, language barriers; lack of access to federal, state, and local policies on health care services; residential segregation; neighborhood disadvantage, marginalization, and stigma (104, 105). Of the 47.9 million immigrants in the country in September, 18.5 million were unemployed. This certainly correlates with immigrants having lower rates of health insurance, less access to health care, and ultimately receiving a lower quality of care than US-born populations. Furthermore, it is estimated that immigrants from Latin American countries other than Mexico represent about 60 percent and undocumented immigrants account for 61 percent (approximately 1.8 million) of the growth in the foreign-born population since January 2021 (101). Unfortunately, the number of undocumented immigrants is likely to continue to grow, given the current restrictions and delays in immigration policy which were aggravated in recent years and during the pandemic. In addition, deportation policies in the US may influence undocumented immigrants and their families hesitant to seek medical care. Many immigrants are relatively young and healthy when arriving to the US to work, and there is evidence of better health outcomes than their U.S.-born counterparts. However, immigrants’ health worsens over time, likely due to acculturation and poor access to care (56). Health policies at a local and federal level can help address the factors that increase these inequities in CV, oncological, and cardio-oncological care. These can be related at the intersection of health, immigration, and employment laws that ensure access to housing, living wages, education, and healthcare for these vulnerable patients.

## 8 Cardio-oncology disparities in the rural population

As a new subspecialty, cardio-oncology care is localized in urban areas at large academic institutions. This leads to significant disparities in cardio-oncology care for rural populations due to decreased access to tertiary care sites and the benefits of subspecialty care, testing, and clinical trial enrollment. The International Cardio-Oncology Society registry shows 21 countries with national cardio-oncology programs, and 81% of centers are in upper-middle to high-income countries (106). A study of oncologists in the central US showed 67.5% practiced in exclusively urban locations, 11.3% in exclusively rural locations, and 21.1% in both rural and urban locations (107). Per 2010 US Census Bureau data, 19.3% of the population is rural (108) and therefore may not have local access to specialized cardio-oncology care. One study showed that mean travel time for medical care for rural patients is 3 times longer *vs*. urban patients (128.9 min *vs.* 41.5 min, p<0.001) (109). Reduced access to care may then result in worse outcomes. Multiple studies in Europe and the US have demonstrated that both increased geographic distance and travel time were independently associated with worse outcomes (110, 111).

A qualitative study in rural Scotland that explored patients’ perspectives on disparities in oncologic care demonstrated that transportation is a major issue (112). American Community Survey data from 2020 showed that 1.6 million rural households do not have access to cars (113). Given the higher poverty levels and hospital closures in these pockets located in the South, Appalachia, the Southwest, and Alaska, many rural patients do not have access to tertiary care hospitals or specialized cardio-oncology care. It is well-established that close and early collaboration between cardiologists, oncologists, and primary care providers achieves higher rates of cardiac optimization and support of optimal cancer treatment and survival (114). Since rural residents have limited access to specialists, and the burden of managing patients undergoing active cancer treatment falls on the primary care provider or the rural oncologist without specialized training in cardio-oncology, which may result in worse outcomes (115). Furthermore, rural patients are underrepresented in clinical trials, which offer novel treatments essential to high-quality cancer care. Most trials are run at urban academic centers with large catchment areas, and rural patients have decreased interest in clinical trials due to financial and transportation barriers (116). Thus, it is crucial to engage in interinstitutional efforts such as connecting community-based cancer centers in rural areas to larger specialists from large academic centers as a gateway to cardio-oncology care and access to novel treatments and trials. This in turn, could also help with more inclusive recruitment of populations that are typically underrepresented in clinical trials

## 9 Social and financial disparities in cardio-oncology care

Social and financial disparities are multifaceted in the highly complex cardio-oncology patient population. CVD and cancer share many risk factors influenced by the social determinants of health (SDOH). And when these two chronic conditions co-exist, there is a cumulative effect in the disparities in medical care and the economic hardship faced by patients and their families (117). Low socioeconomic status, racial inequities, lack of support, and barriers to accessing quality medical care have been associated with increased death and CV co-morbidities. Due to limited access to insurance and follow-up care, patients from immigrant and underserved groups historically have increased CVD at baseline and have advanced cancer stages at the time of diagnosis, requiring more cardiotoxic regimens and close surveillance with specialized cardio-oncology care (118, 119). However, the geographic availability of cardio-oncology centers is mainly limited to academic institutions in major cities, which tend to be more challenging to access by these patients of lower socioeconomic status and those without health insurance. Even in large academic centers, appointment availability is sparse and patients often wait months prior to being seen by a specialist in cardio-oncology. Additional barriers to access to care and focused surveillance include the availability of transportation and the ability to attend medical appointments relative to employment status and job flexibility, which are often more difficult for patients of lower socioeconomic backgrounds and minority populations. In addition, there is a higher psychological burden related to financial distress linked with cardio-oncology care in these populations due to the inability to pay medical bills, cost-related delayed care, medication non-adherence, and food and job insecurity.

Economic hardship due to chronic illness has long-term consequences, with cancer being one of the most cited reasons for medical cost-associated bankruptcy in the US (120, 121). There are also population-level disparities in equitable access to specialized care and affordable diagnostic procedures and treatments (37, 122). Understanding the intersection of race, ethnicity, and these socioeconomic disparities is crucial. Furthermore, there are institution-level disparities as patients with challenges due to low income or lack of health insurance tend to receive care in public or safety net hospitals with limited specialized services. There is a need to determine quality metrics in the systems of care for cardio-oncology patients within and between institutions and how this relates to SDOH to improve patient outcomes for all communities, particularly those from underrepresented racial, ethnic, and lower socioeconomic backgrounds.

## 10 Strategies and future directions

A multi-pronged approach is critical to address disparities in the abovementioned populations. Additionally, each group has unique challenges that need to be overcome to achieve equity in the delivery of cardio-oncology care. It is critical that community and healthcare-based efforts are started promptly while research-based efforts are continued to find ways to ensure sustained and long-term equity. Some solutions for improvement on a community and healthcare level include expanding government-sponsored insurance and support programs nationwide to help facilitate access to high-quality and specialized cardio-oncology centers. This can potentially assist at-risk and underserved populations in addressing financial barriers such as coverage for diagnostic studies, medications, and services, as well as reduce issues surrounding care access such as transportation, missed workdays, and childcare. It is vital to design studies and interventions that define, screen for, and mitigate the financial consequences of cardio-oncology care through financial navigation plans. However, further investigation is needed to develop effective policies and methods at a system level focused on value-based care and lower financial burden on cardio-oncology patients, as well as to better understand the unique challenges faced by underrepresented and underserved populations in accessing cardio-oncology care.

While some studies have assessed the socioeconomic factors influencing financial hardship in patients (123–125), there is a need to move toward integrating specific methods and policies at a system level. Furthermore, patients from underrepresented and underserved populations, such as immigrants that could be non-English speakers or undocumented, face heightened challenges that require adequate assistance to fully understand the complex medical and financial issues in accessing cardio-oncology care. Promoting a diversified physician workforce and engaging community health workers with language and cultural experience can help bridge the existing gap and provide guidance to culturally specific resources available to these communities.

It is also key to increase awareness of the multiple social and financial inequities in cardio-oncology care. Advocacy efforts from stakeholders are crucial to developing pathways that provide optimal care while supporting patients in these areas of inequity. Addressing SDOH and the financial toxicity due to chronic CV and cancer care can help improve patient outcomes and enable the participation of underserved minorities in clinical trials in cardio-oncology.

## Author contributions

Study conception and design: SP, GS-A, and NF; data collection, analysis, and interpretation of results: SP, GS-A, AA, and MR; draft manuscript preparation: SP, GS-A, AA, MR, and NF. All authors contributed to the article and approved the submitted version.
